# Antidepressant prescriptions by provider in patients with kidney failure and depression

**DOI:** 10.1093/ckj/sfaf374

**Published:** 2025-12-05

**Authors:** Dong Hui Shin, Deok Gie Kim, Sung Hwa Kim, Tae Sic Lee, Sang Won Hwang, Jun Young Lee, Jinhee Lee

**Affiliations:** Department of Nephrology, Comprehensive Kidney Disease Research Institute, Yonsei University Wonju College of Medicine, Seoul, South Korea; Department of Surgery, The Research Institute for Transplantation, Yonsei University College of Medicine, Wonju, South Korea; Department of Statistics, Yonsei University Wonju College of Medicine, Wonju, South Korea; Department of Convergence Medicine, Yonsei University Wonju College of Medicine, Wonju, South Korea; Department of Precision Medicine, Yonsei University Wonju College of Medicine, Wonju, South Korea; Department of Nephrology, Comprehensive Kidney Disease Research Institute, Yonsei University Wonju College of Medicine, Seoul, South Korea; Department of Psychiatry, Yonsei University Wonju College of Medicine, Wonju, Korea

**Keywords:** antidepressive agents, cohort study, depression, end-stage kidney disease, mortality

## Abstract

**Background:**

The prevalence of depression is high among patients with end-stage kidney disease (ESKD). Recent studies have indicated under-recognition and -treatment of depression in this population, and little is known about how the specialty of the prescribing clinician may influence clinical outcomes. This study aimed to evaluate whether the prescribing clinician’s specialty (psychiatrist vs. non-psychiatrist) is associated with clinical outcomes in patients with ESKD and comorbid depression who receive antidepressant treatment.

**Methods:**

We extracted data from the Korean National Health Institute Database System from January 2004 to December 2022. Patients with ESKD and depression who underwent antidepressant therapy after their ESKD diagnosis were included. Patients were followed up for 4.7 ± 3.5 years.

**Results:**

Among 16 756 patients with ESKD and depression [mean age, 67.3 years; 8614 (51.4%) men], 7841 (46.8%) patients were prescribed antidepressants by psychiatrists. After propensity score matching, the 5-year mortality was significantly lower in the psychiatrist (25.8%) than in the non-psychiatrist group (38.2%). After multivariable adjustment, prescription by a psychiatrist remained significantly associated with lower mortality (adjusted hazard ratio, 0.66; 95% confidence interval, 0.62–0.70; *P* < .001). All-cause mortality was consistent across various subgroups, such as age (above or below 75 years), sex, time from dialysis initiation to depression diagnosis, income level, region of residence, and comorbidity status. This trend remained in 6-month, 1-year, 2-year, and 3-year landmark analyses.

**Conclusions:**

Our findings suggest a potential benefit of specialty psychiatric care for improving clinical outcomes in patients with ESKD and depression.

KEY LEARNING POINTS **What was known:**

- Many people with end-stage kidney disease also experience depression.
- However, not all patients receive antidepressants from psychiatric specialists.

**This study adds:**

- In this nationwide large Korean cohort study of 16 756 patients with both end-stage kidney disease and depression, those who were treated with antidepressants by psychiatrists had a lower 5-year death rate (25.8%) compared to those treated by non-psychiatrists (38.2%).

**Potential impact:**

- These findings suggest that seeing a psychiatrist for depression treatment may help improve survival in patients with serious kidney disease.

## INTRODUCTION

Depression is the most common psychiatric health condition in patients with end-stage kidney disease (ESKD). The prevalence of depression in patients with ESKD is 20%–40%, which is 3–4 times higher than in the general population [1–3]. Several studies have reported that in patients with ESKD, depression is associated with an increased rate of hospitalization and mortality [4–6]. Therefore, depression treatment in patients with ESKD has the potential to modify other clinical outcomes, including survival [7].

However, the quality of depression treatment may be influenced by who prescribes the medication. For instance, a multi-center study from the UK reported that among hemodialysis patients prescribed antidepressants, most received their prescriptions from general physicians, with only ∼10% being managed by psychiatrists. However, the clinical outcomes associated with these different prescribing [8] patterns remain unexplored.

When prescribing antidepressants to patients with ESKD, who are vulnerable to cardiovascular disease, it is essential to consider cardiovascular side effects and dosage [3, 9, 10]. Therefore, it is important that the prescription is made by specialists, such as psychiatrists, with extensive experience in managing these medications [11]. To date, no study has compared the effectiveness of antidepressant prescriptions between psychiatrists and non-psychiatrists among patients with ESKD.

Therefore, we used data from the Korean National Health Insurance Service (NHIS) to compare the prognosis and prescription patterns of patients with ESKD with depression who received an antidepressant prescription through a psychiatry department versus those who received it through a non-psychiatric department.

## MATERIALS AND METHODS

### Data source and study population

This study used data from the NHIS database of South Korea. The NHIS is a mandatory national health insurance program operated by the Korean government, covering ∼97% of the population. The NHIS database has been extensively validated and employed in numerous studies. Data were accessed via the Korean National Health Insurance Sharing Service (http://nhiss.nhis.or.kr) with the appropriate approval (NHIS-2021-1-343). Details of diagnostic and medication codes used in this study are provided in Supplementary data, Tables S1 and S2. In addition, there was a separate set of billing codes specifically designated for psychiatric consultations (see Supplementary data, Table S3), which can only be used by psychiatrists. Using these codes, we classified patients receiving antidepressant medications into two groups: a psychiatrist-managed group (PSY group) comprising patients prescribed antidepressants through psychiatric consultations, and a non-psychiatrist-managed group (non-PSY group) comprising patients prescribed antidepressants by non-psychiatrist physicians.

We initially identified 537 854 patients diagnosed with ESKD in the NHIS database between 1 January 2004 and 31 December 2023. From this cohort, we excluded patients aged under 18 years (*n* = 7575), those who did not undergo regular laboratory examinations provided by the National Health Screening Program (n = 90 297), those diagnosed with depression before the ESKD diagnosis (*n* = 203 907), and those who received antidepressant treatment for <90 consecutive days (*n* = 219 319). As a consequence, a total of 16 576 patients were included in the analysis, consisting of 8913 patients in the PSY group and 7841 patients in the non-PSY group.

**Figure 1:**
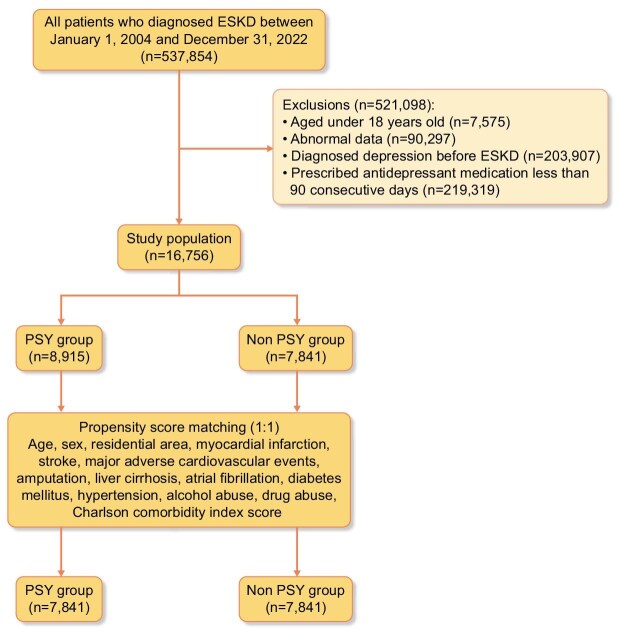
Flow diagram showing selection of the study population.

To compare outcomes between the PSY and non-PSY groups, we employed 1:1 propensity score matching (PSM) using the nearest neighbor method without replacement, with a caliper width of 0.05 standard deviations of the logit of the propensity score. Propensity scores were calculated based on age, sex, residential area, myocardial infarction, stroke, major adverse cardiovascular events (MACE), amputation, liver cirrhosis, atrial fibrillation, diabetes, hypertension, alcohol abuse, drug abuse, and Charlson comorbidity index score (Fig. 1). Covariate between before and after matching was assessed by calculating the standard mean difference, with values <0.2 indicating acceptable balance between groups (Supplementary data, Fig. S2).

The study was approved by the Institutional Review Board of Yonsei University Wonju College of Medicine (Wonju, Korea; approval number CR324339). The requirement for written informed consent was waived because the analysis involved anonymized and de-identified patient data.

### Statistical analysis

Baseline characteristics were compared between the PSY and non-PSY groups both before and after PSM using the *t*-test for continuous variables and Chi-square test for categorical variables. Continuous variables are expressed as mean ± standard deviation, while categorical variables are presented as numbers and percentages. To mitigate immortal time bias, we applied the landmark analysis method (Supplementary data, Fig. S1). The landmark date was defined to ensure that all patients were followed for at least 1 year, with the last cohort entry date set to 31 December 2018. Patients who died between cohort entry and the landmark date were excluded from the analysis. Participants who prescribed antidepressant medication by psychiatrists within the landmark period were categorized into the PSY group, whereas those who prescribed by psychiatrists after the landmark period or not at all were assigned to the non-PSY group.

Kaplan–Meier survival curves and log-rank tests were used to compare the cumulative incidences of outcomes between the matched groups. After matching, hazard ratios (HRs) were estimated using multivariate Cox regression models, adjusting for baseline covariates that showed significant differences between groups before matching. HRs were further adjusted for variables that remained significantly different between groups after PSM. In addition, according to the QT-prolong potential of selective serotonin reuptake inhibitors (SSRIs), we compared the proportion of prescriptions and clinical outcomes between higher and lower QT-prolong potential SSRI groups [12]. All statistical analyses were conducted using R statistical software (version 4.2.0 for Windows, http://cran.r-project.org). A *P* value of <.05 was considered to indicate statistical significance.

## RESULTS

### Baseline characteristics

Before matching, 8915 patients [mean (SD) age, 67.3 (12.9) years; 4436 (49.8%) men] received antidepressants prescribed by psychiatrists [PSY group) and 7841 (mean (SD) age, 66.2 (13.3); 4175 (53.3%) men] received antidepressants prescribed by non-psychiatrists (non-PSY group). The non-PSY group had a shorter follow-up period than the PSY group (4.0 ± 3.3 vs. 5.2 ± 3.6 years). Furthermore, the non-PSY group had significantly more comorbidities, such as history of myocardial infarction (21% vs. 20.0%), stroke (51.9% vs. 44.3%), atrial fibrillation (17.3% vs. 15.3%), diabetes (91.4% vs. 89.2%), and hypertension (98.7% vs. 97.5%) than the PSY group. The non-PSY group had a higher Charlson comorbidity index score than the PSY group (7.9 ± 3.0 vs. 5.6 ± 3.0). The detailed baseline characteristics of the two groups before and after matching are shown in Supplementary data, Tables S4 and S5. After PSM, all variables were similar between the PSY and non-PSY groups (Table 1). The standard mean differences before and after matching are shown in Supplementary data, Fig. S2.

**Table 1:** Baseline characteristics after match.

Variables	Total (*N* = 15 682)	Non-PSY (*N* = 7841)	PSY (*N* = 7841)	Standard mean difference
Age	66.9 ± 12.9	67.6 ± 12.5	66.2 ± 13.3	0.153
Sex	8 221 (52.4)	4 046 (51.6)	4 175 (53.3)	0.019
ESKD ∼ depression (year)	1.8 ± 2.6	2 ± 2.7	1.7 ± 2.4	0.069
Interval of follow-up period	4.7 ± 3.5	4.1 ± 3.3	5.2 ± 3.6	
Year of ESKD				
2004–2009	4 650 (29.6)	2 282 (29.1)	2 368 (30.2)	
2010–2015	5 489 (35.0)	2 737 (34.9)	2 752 (35.1)	
2016–2022	5 544 (35.4)	2 823 (36.0)	2 721 (34.7)	
Income level				0.046
Quantile 1	4 440 (28.3)	2 245 (28.6)	2 195 (28)	
Quantile 2	1 996 (12.7)	1 003 (12.8)	993 (12.7)	
Quantile 3	3 091 (19.7)	1 549 (19.8)	1 542 (19.7)	
Quantile 4	6 155 (39.3)	3 044 (38.8)	3 111 (39.7)	
Residential area				0.033
Rural	8 010 (51.1)	4 057 (51.7)	3 953 (50.4)	
Urban	7 672 (48.9)	3 784 (48.3)	3 888 (49.6)	
Myocardial infarction	3 184 (20.3)	1 619 (20.7)	1 565 (20)	0.027
Stroke	7 208 (46)	3 735 (47.6)	3 473 (44.3)	0.017
Atrial fibrillation	2 484 (15.8)	1 281 (16.3)	1 203 (15.3)	0.016
Diabetes mellitus	14 112 (90)	7 118 (90.8)	6 994 (89.2)	0.027
Hypertension	15 373 (98)	7 728 (98.6)	7 645 (97.5)	0.053
Charlson comorbidity index score	6.8 ± 3.1	6.9 ± 3.1	6.7 ± 3.1	0.108

### Clinical outcomes

The 1-, 3-, and 5-year cumulative incidences of all-cause mortality were 15.1%, 28.7%, and 36.3% in the non-PSY group and 7%, 18.3%, and 25.8% in the PSY group, respectively (Supplementary data, Table S6). Except for unknown cause, the most common cause of mortality in both groups was cardiovascular disease (CVD), followed by infection and cancer. Compared to the PSY group, the non-PSY group had a higher proportion of death due to CVD (20.9% vs. 18.8%, *P* < .001). Conversely, death due to suicide were more common in the PSY group (1.7% vs. 1.0%, *P* < .001; Table 2).

**Table 2:** Cause of death of two groups.

	Non-PSY group	PSY group	*P*
Unknown	1677 (36.5)	1517 (34.7)	.284
Cardiovascular disease	961 (20.9)	825 (18.8)	<.001
Infection	521 (11.4)	516 (11.8)	.519
Cancer	472 (10.3)	493 (11.3)	.135
Gastrointestinal disease	126 (2.7)	153 (3.5)	.041
Pulmonary disease	74 (1.6)	72 (1.6)	.904
Psychiatric disease	65 (1.4)	110 (2.5)	.378
Suicide	44 (1.0)	75 (1.7)	<.001
Other	694 (15.1)	692 (15.8)	.384

**Figure 2:**
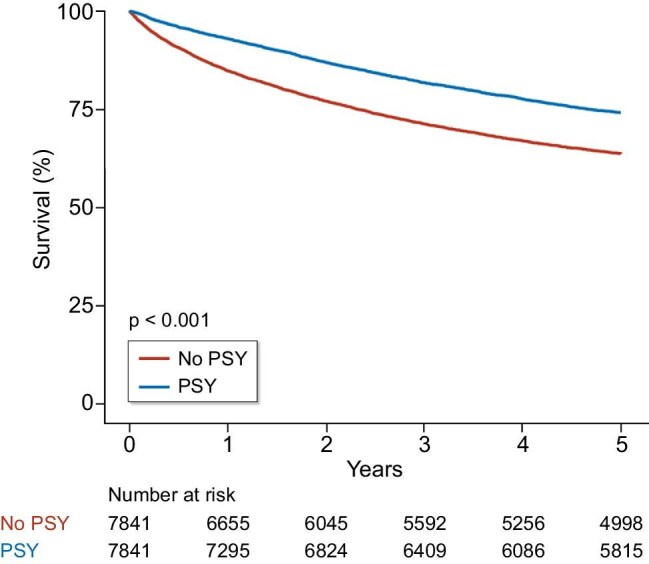
Kaplan–Meier curve for all-cause death.

All-cause mortality was significantly lower in the PSY group than in the non-PSY group (*P* *< *.001) based on Kaplan–Meier curve analysis (Fig. 2). According to the multivariate Cox regression analysis (no landmark point), the HR for mortality in the PSY group was significantly lower [0.66; 95% confidence interval (CI): 0.62–0.70, *P* *< *.0001] than that in the non-PSY group. Using landmark analysis and multivariate Cox regression analysis at 6 months, the PSY group maintained a significantly lower HR of 0.73 (95% CI: 0.69–0.78, *P* *< *.0001). Similarly, at 1 year, the HR was 0.81 (95% CI: 0.69–0.86, *P* *< *.0001); at 2 years, it was 0.77 (95% CI: 0.75–0.84, *P* *< *.0001); and at 3 years, it remained significantly lower at 0.71 (95% CI: 0.63–0.80, *P* *< *.0001) (Table 3). These findings consistently indicated a significantly reduced risk of mortality in the PSY group compared to that in the non-PSY group at various landmark time points (Supplementary data, Fig. S3). The univariate analysis results of those outcomes were similar (Supplementary data, Table S7).

**Table 3:** Risk of death with landmark analysis and without landmark analysis (multivariate analysis).

		HR (95% CI)	*P*
Landmark-no		0.66 (0.62–0.70)	<.001
Landmark-yes	6 months	0.73 (0.69–0.78)	<.001
	1 year	0.81 (0.69–0.86)	<.001
	2 years	0.77 (0.75–0.84)	<.001
	3 years	0.71 (0.63–0.80)	<.001

**Figure 3:**
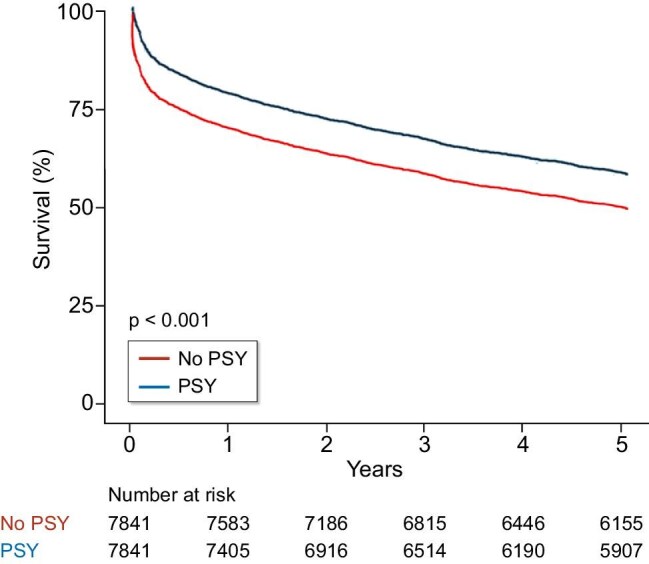
Kaplan–Meier curve for MACE.

Subgroup analyses stratified by age, sex, duration from ESKD to depression diagnosis, income, hypertension, diabetes, residential area, Charlson comorbidity index score, history of drug abuse, and type of antidepressant prescription consistently demonstrated a greater reduction in mortality risk in the PSY group than in the non-PSY group (Table 4). In addition, compared to the non-PSY group, the risk of MACEs was significantly lower in the PSY group than in the non-PSY group (HR: 0.71; 95% CI: 0.66–0.71; Fig. 3).

**Table 4:** Subgroup analysis of all-cause mortality according to patient characteristics.

	*N*	HR (95% CI)	*P* value	Interaction *P*
Age ≥75	10 869	0.68 (0.63–0.74)	<.001	0.564
Age <75	4813	0.63 (0.58–0.68)	<.001	
Male	8221	0.70 (0.65–0.76)	<.001	0.344
Female	7461	0.60 (0.55–0.65)	<.001	
ESKD to depression ≥1 years	9016	0.68 (0.62–0.74)	<.001	0.456
ESKD to depression <1 years	6666	0.61 (0.57–0.65)	<.001	
High income	11 242	0.59 (0.53–0.66)	<.001	0.669
Low income	4440	0.67 (0.62–0.71)	<.001	
No hypertension	309	0.65 (0.62–0.69)	<.001	0.755
Hypertension	15 373	0.42 (0.24–0.74)	.003	
No diabetes	1570	0.65 (0.61–0.69)	<.001	0.643
Diabetes	14 112	0.70 (0.54–0.90)	.005	
Urban area living	8010	0.61 (0.56–0.66)	<.001	0.523
Rural area living	7672	0.68 (0.63–0.73)	<.001	
CCI ≥5	11 896	0.74 (0.67–0.82)	<.001	0.433
CCI <5	3786	0.61 (0.57–0.66)	<.001	
No drug abuse	13 466	0.68 (0.58–0.79)	<.001	0.776
Drug abuse	2216	0.64 (0.60–0.68)	<.001	
SSRIs yes	4677	0.68 (0.63–0.74)	<.001	0.120
SSRIs no	11 005	0.73 (0.67–0.80)	<.001	
TCA yes	7201	0.62 (0.55–0.71)	<.001	0.150
TCA no	8481	0.69 (0.65–0.74)	<.001	

Abbreviation: CCI, Charlson comorbidity index.

When examining prescribed medications, we observed that the PSY group received significantly more prescriptions for SSRIs (25.5% vs. 37.8%, *P* *< *.001), serotonin antagonist reuptake inhibitors (9.5% vs. 12.8%, *P* *< *.001), noradrenergic and specific serotonin antidepressants (2.2% vs. 5.8%, *P* *< *.001), and monoamine oxidase inhibitors (0% vs. 0.1%, *P* *= *.039) than the non-PSY group. Meanwhile, the non-PSY group received significantly more prescriptions for serotonin-norepinephrine reuptake inhibitors (10.3% vs. 8.1%, *P* *= *.024) and tricyclic antidepressants (TCAs) (58.5% vs. 33.4%, *P* *< *.001; Supplementary data, Table S8) than the PSY group. Although the absolute number of QT-prolonging SSRIs remained higher in the PSY group, psychiatrists prescribed these agents (citalopram and escitalopram) significantly less frequently than non-psychiatrists (Supplementary data, Table S9). Notably, compared with TCAs the short-QT SSRI subgroup (sertraline, paroxetine, fluoxetine, fluvoxamine) showed a lower risk of all-cause death [HR 0.64 (95% CI, 0.58–0.71)] than the long-QT SSRI subgroup (citalopram, escitalopram; HR 0.71 [95% CI, 0.65–0.78)], with a significant interaction (*P* for interaction, .03) (Supplementary data, Table S10).

## DISCUSSION

To the best of our knowledge, this study is the first to reveal that in patients with ESKD and depression, antidepressant prescription by psychiatrists is associated with a significantly higher survival than antidepressant prescription by non-psychiatric physicians. The results were consistent in PSM and landmark analyses. The observed survival benefit in the psychiatrist-prescribed group could be interpreted as the result of more accurate diagnostic assessments, patient-specific medication selection and dose adjustment, and the continuous and systematic treatment monitoring provided by psychiatric specialists.

This finding is particularly significant in the context of real-world clinical practice, where specialist involvement is often limited. For instance, a small-scale UK study of patients on hemodialysis reported that psychiatrists were responsible for prescribing antidepressants in only 10% of cases, underscoring the suboptimal patterns of depression management in this population [8]. Several studies have emphasized that depression in patients with ESKD is associated with reduced survival rates [4, 5, 7, 13]. However, there is limited research on the impact of the prescribing physician’s specialty on patient outcomes following antidepressant treatment [14–16]. In general, there are relatively few studies comparing depression treatment provided by psychiatrists versus non-psychiatric physicians, making it difficult to determine which treatment approach is superior [17, 18]. However, consistent with our findings, compared to non-psychiatric physicians, the treatments provided by trained psychiatrists tended to differ in terms of medication selection and often included additional therapeutic approaches, such as psychotherapy, resulting in better depression control [8, 16, 18, 19]. Although few studies have directly evaluated the impact of psychiatric treatment on survival rates and no study has compared those effects among patients with ESKD, it is reasonable to assume that the appropriate treatment of psychiatric conditions could significantly influence patient outcomes and prognosis, given the higher mortality rates among patients with mental disorders [20]. Furthermore, our results indicate that extensive experience with antidepressants treatment, such as that of psychiatrists, may contribute to better survival outcomes than the limited experience with antidepressant prescriptions of non-psychiatric physicians.

In addition, our subgroup analysis based on the QT-prolonging potential of SSRIs provided further insight into the mechanisms underlying the survival advantage in the psychiatrist-treated group. Although the absolute number of SSRIs with higher QT-prolonging potential remained greater among patients treated by psychiatrists, these agents (citalopram and escitalopram) were prescribed significantly less frequently than by non-psychiatrists. When compared with TCAs, SSRIs with lower QT-prolong potential (sertraline, paroxetine, fluoxetine, fluvoxamine) were associated with a lower risk of all-cause death (HR; 0.64 95% CI: 0.58–0.71) than those with the long-QT-prolong potential (HR; 0.71 95% CI: 0.65–0.78), with a significant interaction (p for interaction = 0.03). These findings suggest that psychiatrists’ selective avoidance of SSRIs with higher QT-prolonging potential may partially explain the improved survival outcomes, possibly by reducing the risk of sudden cardiac death [12].

In patients with ESKD experiencing depression, the involvement of psychiatrists may offer a significant advantage that contributes to improved survival rates. For example, psychiatrists possess specialized training that enables the precise evaluation of depression severity in patients with ESKD. This accuracy is crucial for formulating effective treatment plans tailored to the individual’s need. They are also adept at selecting suitable antidepressant medications and adjusting dosages appropriately, considering the unique physiological challenges posed by ESKD [21–23]. This expertise helps in minimizing potential side effects and enhancing medication adherence. For instance, certain antidepressants require careful dosing adjustments to avoid adverse effects in patients with ESKD, underscoring the importance of specialist involvement. Beyond pharmacotherapy, psychiatrists can integrate non-pharmacological treatments, such as cognitive–behavioral therapy, into the care plan. Cognitive–behavioral therapy has been shown to effectively reduce depressive symptoms and improve quality of life in patients with ESKD, thereby potentially enhancing the survival rate [9].

Psychiatric disorders, including depression, are associated with CVDs, such as hypertensive disorders, ischemic heart disease, angina pectoris, stroke, and venous thromboembolism [24, 25]. Stress-induced activation of the hypothalamic-pituitary-adrenal axis and alterations in sympathetic nervous system activity, both of which are heightened following psychiatric disorders, directly contribute to elevated blood pressure, heart rate instability, and accelerated atherosclerosis [26]. Furthermore, the psychological burden plays a crucial role in the destabilization of atherosclerotic plaques, which are related to cardiovascular events [27].

Appropriate antidepressant treatment can improve endothelial function and reduce arterial stiffness, thereby potentially lowering the risk of coronary artery disease [28, 29]. A British cohort study reported that in the general population, some antidepressants (for example, fluoxetine) were associated with a decreased risk of CVD, whereas others (for example, lofepramine) were associated with an increased risk of CVD [30]. In patients with ESKD, who are particularly vulnerable to cardiovascular complications, a previous cohort study suggested that proper management of depression may be associated with a reduced cardiovascular risk [31]. However, to date, no study has directly compared clinical outcomes, such as all-cause mortality or MACE according to the type of antidepressant used. Further research is needed to guide individualized antidepressant selection in patients with ESKD.

This study has some limitations. First, due to the nature of the data from the NHIS, detailed information, such as the depression severity, education level, alcohol consumption, smoking, and individual psychosocial factors, was missing. Thus, we could not completely eliminate selection bias related to these parameters. Second, because of the nature of nationwide data, obtaining laboratory values of hemoglobin and albumin was impossible, making it difficult to assess comorbidities. Third, the diagnosis of cardiovascular, cerebrovascular, and psychiatric diseases was established through an operational definition, which may have led to misdiagnosis. Fourth, as healthcare practices and insurance systems differ substantially across countries, the generalizability of our findings beyond South Korea is limited. In Korea, both drug prices and medical service fees are strictly regulated by the national health insurance system, making antidepressant prescriptions more accessible and less affected by patients’ socioeconomic status compared with many other countries. Therefore, the results should be interpreted within the context of the Korean health care environment. Last, as this was a cohort study, causal relationships could not be established. Therefore, further prospective studies are needed to confirm the findings.

In conclusion, this nationwide observational cohort study demonstrated that, among patients with ESKD and comorbid depression, antidepressant treatment provided by psychiatrists was associated with a reduced risk of all-cause mortality and MACE compared with antidepressant treatment provided by non-psychiatrist physicians. Although receiving psychiatric care may be challenging for some patients with ESKD, individualized antidepressant treatment by trained mental health specialists appears to play a crucial role in improving outcomes in this population.

## Supplementary Material

sfaf374_Supplemental_File

## Data Availability

Data are available with the approval and oversight of the NHIS (NHIS-2024-1-520) through the Korean National Health Insurance Sharing Service.
