# Supplementary material for: Antidepressant prescriptions by provider in patients with kidney failure and depression
Source: Clin Kidney J. 2025 Dec 5;19(1):sfaf374. doi: 10.1093/ckj/sfaf374 (PMC12813503; doi:10.1093/ckj/sfaf374)

**Antidepressant prescriptions by provider in patients with end-stage kidney disease and depression**

Running head: Antidepressant in end stage kidney disease

Dong Hui Shin^1,*^, Deok Gie Kim^2*^, Sung Hwa Kim^3^, Tae Sic Lee^4^, Sang Won Hwang^5^, Jun Young Lee^1^, Jinhee Lee^6^

^1^ Department of Nephrology, Comprehensive Kidney Disease Research Institute, Yonsei University Wonju College of Medicine, ^2^ Department of Surgery, The Research Institute for Transplantation, Yonsei University College of Medicine, ^3^ Department of Statistics, Yonsei University Wonju College of Medicine, ^4^ Department of Convergence Medicine, Yonsei University Wonju College of Medicine, ^5^ Department of Precision Medicine, Yonsei University Wonju College of Medicine, ^6^ Department of Psychiatry, Yonsei University Wonju College of Medicine, Wonju, Korea

*These authors contributed equally to this work.

^*^Corresponding Authors:

Jun Young Lee, MD, PhD, Department of Nephrology, Yonsei University Wonju College of Medicine, Wonju 26426, Korea.

Email: [junyoung07@yonsei.ac.kr](mailto:junyoung07@yonsei.ac.kr)

Jinhee Lee, MD, PhD, Department of Psychiatry, Yonsei University Wonju College of Medicine, Wonju, Korea

Email: [jinh.lee95@yonsei.ac.kr](mailto:jinh.lee95@yonsei.ac.kr)

**SUPPLEMENTARY MATERIAL**

Table of Contents

**Table S1:** Diagnoses, procedures, and specific codes.

**Table S2:** Drugs (code and dose) reported in the National Health Insurance Korea database analysis.

**Table S3:** Specific code of individualized psychotherapy.

**Table S4:** Detailed baseline characteristics of the patients before matching.

**Table S5:** Detailed baseline characteristics of patients after matching.

**Table S6:** Cumulative incidence and hazard ratio of death before and after matching.

**Table S7**: Risk of death with landmark analysis and without landmark analysis (univariate analysis).

**Table S8**: Type of prescribed antidepressants between two group (before and after matching).

**Table S9:** Type of prescribed selective serotonin reuptake inhibitors (SSRIs) between two groups.

**Table S10:** Type of prescribed selective serotonin reuptake inhibitors (SSRIs) and clinical outcomes.

**Figure S1.** Graphic depiction of landmark analysis.

**Figure S2**. Standard mean difference before and after matching.

**Figure S3**. Kaplan-Meier curve for all cause death (landmark analysis).

**Table S1:** Diagnoses, procedures, and specific codes.

| **Disorder** | **Diagnosis, procedure, specific codes*** |
| --- | --- |
| Acquired immunodeficiency syndrome | B20 B21, B22, B24 |
| Alcohol abuse | F10, E52, G62.1, I42.6, K29.2, K70.0, K70.3, K70.9, T51.x, Z50.2, Z71.4, Z72.1 |
| Amputation | N0573, N0574, N0571-5, N0562, N0564, N0565, N0566, Y835 |
| Atrial fibrillation | I480, I482, I4891 |
| Cancer | C00 to C97 |
| Cerebrovascular disease | G45, G46, I60, I61, I62, I63, I64, I65, I66, I67, I68, I69, H340 |
| Chronic lung disease | J40, J41, J42, J43, J44, J45, J46, J47, J60, J61, J62, J63, J64, J65, J66, J67, I278, I279,  J684, J701, J703 |
| Diabetes | E11 to E14 or ≥ 1 antidiabetic medication |
| Dementia | F00, F01, F02, F03, G30, G311, F051 |
| Depression | F32.x, F33.x, F34.1 |
| Drug Abuse | F11.x - F16.x, F18.x, F19.x, Z71.5, Z72.2 |
| Heart failure | I43, I50, I099, I110, I130, I132, I255, I420, I425, I426, I427, I428, I429, I97.1, P290 |
| Hemiplegia | G81, G82, G041, G114, G801, G802, G830, G831, G832, G833, G834, G839 |
| Hemodialysis | Z491, N185, I120, Z992, O7020, O7021, V001* |
| Hypertension | I10, I11, I12, I13, I15 or ≥ 1 antihypertensive medication |
| Liver cirrhosis | K74.60 |
| MACE | Composite of cardiovascular mortality, acute myocardial infarction, and stroke |
| Malignancy without metastasis | C00 to C97 (except C77, C78, C79, C80) |
| Metastatic solid tumor | C77, C78, C79, C80 |
| Mild liver disease | B18, K73, K74, K700, K701, K702, K703, K709, K713, K714, K715, K717, K760,  K762 K763, K764, K768, K769, Z944 |
| Moderate-to-severe liver disease | I850, I859, I864, I982, K704, K711, K721, K729, K765, K766, K767, K704, K711 |
| Myocardial infarction | I21, I22, I252 |
| Peptic ulcer disease | K25, K26, K27, K28 |
| Peripheral vascular disease | I700 to I702, I708, I709, K551, K558, K559, Z958, Z959, I1731, I1738, I1739, I1771,  I1790, I1792 |
| Peritoneal dialysis | Z492, O7061, O7062, O7071, O7072, O7074, O7076, O7077, V003* |
| Rheumatologic disease | M05, M06, M32, M33, M34, M315, M351, M353, M360 |
| Vascular disease | I20, I21, I22, I24, I25 |
| Stroke | I60–63 and admitted more than two days |
| Suicide attempt | X60–84, Y87, R458, z915 |

All diagnostic, procedural, and specific codes are available at htt[ps://www](http://www.hira.or.kr/).hir[a.o](http://www.hira.or.kr/)r[.kr/.](http://www.hira.or.kr/) Abbreviations: MACE, major adverse cardiovascular event

*Specific code: With this code, patients on dialysis can receive a certificate of disability and an additional insurance discount.

In this study, the requested and approved NHIS data were merged with the data from the mortality records database of Statistics

Korea ([http://mdis.kostat.go.kr](http://mdis.kostat.go.kr/)), which includes the cause and date of mortality. ICD-10 codes were used in the analyses.

**Table S2:** Drugs (code and dose) reported in the National Health Insurance Korea database analysis.

| **Drug** | **Health Insurance Review & Assessment (HIRA) service code** |
| --- | --- |
| ACEi or ARBs or aliskiren | 104201ATB, 104202ATB, 114701ATB, 122601ATB, 122602ATB, 122603ATB, 122901ATB, 122902ATB, 122903ATB, 133001ATB, 133002ATB, 133003ATB,  140901ATB, 140902ATB, 151601ATB, 151603ATB, 163501ATB, 163502ATB, 104201ATB, 104202ATB,114701ATB, 122601ATB, 122602ATB, 122603ATB, 122901ATB, 122902ATB, 122903ATB, 133001ATB, 133002ATB, 133003ATB, 140901ATB, 140902ATB, 151601ATB, 151603ATB, 163501ATB, 163502ATB, 173401ATB, 173402ATB, 177301ATB, 177303ATB, 177303ATB, 184501ATB, 185701ATB, 185702ATB, 196801ATB, 196802ATB, 211301ATB, 211302ATB, 221901ATB, 222401ACH, 222401ATB, 222402ACH, 222402ATB, 222404ATB, 235002ATB, 247101ATB, 247102ATB, 247103ATB, 247104ATB, 378801ATB, 378802ATB, 378803ATB, 429201ATB, 468501ATB, 468502ATB, 468503ATB, 501601ATB, 501602ATB, 510401ATB, 510402ATB, 510403ATB, 515201ATB, 515202ATB, 515203ATB, 520901ATB, 520902ATB, 662401ATB, 662402ATB, 662403ATB, 651401ATB, 651402ATB, 651403ATB |
| BBs | 117903ATB, 117904ATB, 124801ATB, 219901ATB, 219902BIJ, 219904ATB, 219905ACR, 219906ACR, 111401ATB, 111402ATB, 111403ATB, 116801ATB, 116803ATB, 117001ATB, 117002ATB, 125001ATB, 125002ATB, 125003ATB, 125004ACR, 125005ATB, 125006ACR, 125007ACR, 125007ATR, 125008ACR, 125008ATR, 483101ATB, 483102ATB, 489501ATB, 489502ATB, 489503ATB, 662201ATB, 662202ATB, 117901ATB, 117902ATB, 129101ATB, 193802ATB, 194003ATR, 198301ATB, 154401BIJ, 154402BIJ, 154431BIJ, 154430BIJ, 180201ATB, 180201BIJ, 180202BIJ, 180230BIJ, 180231BIJ |
| CCBs | 107601ATB, 107601ATD, 107602ATB, 107602ATD, 107603ATB, 114001ACH, 114002ACH, 114003ACH, 115101ATB, 115102ATB, 115103ATB, 115104ATB, 133101ATB, 133102ATB, 157501ATR, 157503ATR, 178902ACR, 180301ATB, 180302ATB, 180303ATB, 182001ATB, 182002ATB, 188001ATB, 188002ATB, 201001BIJ, 201002ATB, 201002BIJ, 201003ACR, 201030BIJ, 201031BIJ, 201033BIJ, 202401ATB, 202402ACS, 202402ATB, 247601ACR, 247603ATR, 247604BIJ, 247605ATR, 247606ATB, 247607ATB, 459801ACH, 459801ATB, 459802ACH, 459901ATB, 459902ATB, 464601ATB, 470801ATB, 470802ATB, 476201ATB, 479701ATB, 483201ATB, 483202ATB, 489501ATB, 489502ATB, 489503ATB, 145702BIJ, 145703ACR, 145704BIJ, 145706ATB, 145706ATR, 145707ACR, 145707ATB, 145707ATR, 201401ACS, 201401ATB, 201402ATB, 201405ATR, 201407ACS, 201408ATR, 201409ATR, 201702ATB, 201901ATB, 201902BIJ, 201930BIJ, 356201ATB, 356202ATB, 356202ATR, 356203ATR, 441201ATB, 441202ATB, 528201ATR, 528202ATR, 145702BIJ, 247630BIJ, 486501ATB, 486502ATB, 495901ATB, 501701ATB |
| Diuretics | 101501ATB, 101502BIJ, 106901ATB, 163801ATB, 163802BIJ, 163830BIJ, 170801ATB, 174401ATR, 174402ATB, 174403ATB, 231101ATB, 231102ATB, 244701ATB, 262700ATB, 367001ATB, 367002ATB, 451301ATB, 451302ATB |
| ACEi or ARBs and CCBs | 447100ATB, 447200ATB, 466000ATB, 492800ATB, 492900ATB, 495800ATB, 500500ATB, 500600ATB, 582200ATB, 582400ATB, 502700ATB, 503000ATB, 513900ATB, 511500ATB, 511600ATB, 511700ATB, 623100ATB, 521200ATB, 521300ATB, 521400ATB, 644800ATB, 522200ATB, 522300ATB, 522400ATB, 522600ATB, 522700ATB, 522800ATB, 522900ATB, 523000ATB, 523100ATB, 523200ATB, 523300ATB, 523400ATB, 547500ATB, 547600ATB, 547700ATB, 547800ATB, 547900ATB, 548000ATB, 631300ATB, 629400ATB, 629500ATB, 629600ATB, 632800ATB, 632900ATB, 633000ATB, 637400ATB, 637500ATB, 637600ATB, 644800ATB, 651900ATB, 652000ATB, 652100ATB, 652700ATB, 652900ATB, 653000ATB, 653100ATB |
| ACEi or ARBs and statins | 524000ATB, 524100ATB, 527000ATB, 527100ATB, 525000ATB, 525100ATB, 525200ATB, 525300ATB, 629700ATB, 629800ATB, 526300ATB, 526400ATB, 526500ATB, 526900ATB, 644100ATB, 644200ATB, 653200ATB, 629900ATB, 630000ATB, 630100ATB, 630200ATB, 631600ATB, 631700ATB, 634900ATB, 635000ATB, 635100ATB, 635200ATB, 653200ATB, 654600ATB, 654700ATB, 654800ATB, 654900ATB, 655000ATB, 661800ATB, 661900ATB, 662000ATB, 662100ATB, 673700ATB, 688100ATB, 688200ATB, 688300ATB, 688400ATB, 688500ATB |
| BB with diuretics | 262100ATB, 262600ATB, 460200ATB, 469800ATB, 469900ATB, 470000ATB |
| ACEi or ARBs and diuretics | 262200ATB, 262300ATB, 262500ATB, 378900ATB, 440300ATB, 453600ATB, 453700ATB, 486900ATB, 356400ATB, 442600ATB, 385700ATB, 385800ATB, 423700ATB, 440800ATB, 443200ATB, 443300ATB, 502600ATB, 448600ATB, 448700ATB, 460500ATB, 477400ATB, 490100ATB, 497900ATB, 499200ATB, 499300ATB, 513600ATB, 522000ATB, 526800ATB, 556200ATB, 673500ATB, 673600ATB |
| ACEi or ARBs and CCBs and diuretics | 519700ATB, 519800ATB, 519900ATB, 520000ATB, 520100ATB, 662800ATB, 662900ATB, 663000ATB, 663500ATB, 663600ATB, 663700ATB, 663800ATB, 682700ATB, 682800ATB, 682900ATB |
| ACEi or ARBs and CCBs and statins | 663900ATB, 664000ATB, 664100ATB, 664200ATB, 664300ATB, 664400ATB, 671200ATB, 671300ATB, 671400ATB, 671500ATB, 671600ATB, 671700ATB, 677000ATB, 677100ATB, 677300ATB, 677400ATB, 677500ATB, 677600ATB, 686800ATB, 679500ATB, 679600ATB, 679700ATB, 680300ATB, 684300ATB, 684400ATB, 684500ATB, 684600ATB, 684700ATB, 686800ATB, 686900ATB, 690400ATB, 690500ATB, 690600ATB, 690700ATB, 691400ATB, 691500ATB |
| CCBs and statins | 472300ATB, 472400ATB, 472500ATB, 518900ATB, 614500ATB, 673900ATB, 674000ATB, 674100ATB, 678600ATB |
| BBs and CCBs | 262400ATR |
| BBs and statins | 683000ATB, 683100ATB, 683200ATB, 691200ATB |
| Alpha blockers | 149101ATB, 149102ATB, 149104ATR, 483401ACH, 104803ATR, 159001ATB, 234601ACR, 234601ATD, 234601ATR, 234602ACR, 234603ACR, 234603ATD, 234603ATR, 235501ATB, 235502ATB, 235503ATB, 458801ACS, 458801ATB, 504201ACH, 504202ACH, 504202ATB, 504203ACH, 504203ATD, 505801ATB, 505802ATD, 614201ATB, 614202ATB, 614203ATB |
| Statins or ezetimibe or fibrate | 111501ATB, 111502ATB, 111503ATB, 111504ATB, 162401ACH, 162402ACH, 162403ATR, 185801ATB, 216601ATB, 216602ATB, 216603ATB, 216604ATB, 218001ATB, 227801ATB, 227801ATR, 227802ATB, 227803ATB, 227805ATB, 227806ATB, 454001ATB, 454002ATB, 454003ATB, 462201ATB, 470901ATB, 470902ATB, 470903ATB, 471000ATB, 471100ATB, 507800ATB, 502201ATB, 502202ATB, 502203ATB, 502204ATB, 519300ACH, 631400ATB, 631500ATB, 633800ATB, 633900ATB, 634600ATB, 634800ATB, 640700ATB, 640800ATB, 640900ATB, 663400ACS, 679300ACH |
| DM medications | 170101BIJ, 170102BIJ, 170103BIJ, 170130BIJ, 170131BIJ, 170401BIJ, 170402BIJ, 170430BIJ, 170431BIJ, 170502BIJ, 175301BIJ, 175302BIJ, 175304BIJ, 175330BIJ, 175331BIJ, 175332BIJ, 175333BIJ, 441301BIJ, 441302BIJ, 441303BIJ, 441304BIJ, 441305BIJ, 441330BIJ, 441331BIJ, 441332BIJ, 441333BIJ, 441334BIJ, 461801BIJ, 461802BIJ, 461804BIJ, 461830BIJ, 461831BIJ, 461832BIJ, 484901BIJ, 484902BIJ, 484930BIJ, 484931BIJ, 488701BIJ, 488730BIJ, 507401BIJ, 626700BIJ, 626801BIJ, 626802BIJ, 626830BIJ, 626831BIJ, 512101BIJ, 512102BIJ, 512130BIJ, 512131BIJ, 626601BIJ, 626602BIJ, 626630BIJ, 626631BIJ, 639701BIJ, 639702BIJ, 644501BIJ, 644502BIJ, 666700BIJ, 667000BIJ, 527301ATB, 527302ATB, 628201ATB, 628202ATB, 636101ATB, 639800ATR, 641400ATR, 649000ATB, 649100ATB, 649200ATB, 649300ATB, 649400ATB, 649500ATB, 674301ATB, 674302ATB, 100601ATB, 100602ATB, 165402ATB, 165601ACS, 165602ACS, 165602ATB, 165603ATR, 165604ATR, 165701ATB, 165702ATB, 165703ATB, 165704ATB, 165801ATB, 191501ATB, 191502AGR, 191502ATB, 191502ATR, 191503ATB, 191504ATB, 191504ATR, 191505ATR, 249001ATB, 249002ATB, 249002ATD, 348002ATB, 379501ATB, 379502ATB, 379503ATB, 406201ATB, 406202ATB, 421100ATB, 430201ATB, 430202ATB, 430203ATB, 431901ATB, 431902ATB, 443400ATB, 443500ATB, 452700ATB, 452900ATB, 469100ATB, 471900ATB, 474200ATB, 474300ATB, 474300ATR, 488800ATB, 488900ATB, 489000ATB, 498100ATB, 498600ATB, 486101ATB, 497200ATB, 498100ATB, 523600ATB, 523700ATB, 525500ATB, 525600ATB, 525901ATB, 631900ATB, 632100ATB, 637200ATB, 653800ATR, 653900ATR, 654000ATR, 655700ATR, 518800ATB, 500801ATB, 501101ATB, 501102ATB, 501103ATB, 502200ATB, 502300ATB, 502300ATR, 502900ATB, 513700ATB, 513700ATR, 524700ATR, 507000ATB, 507100ATB, 519600ATB, 518500ATR, 518600ATR, 520500ATB, 520600ATB, 520700ATB, 523800ATR, 632000ATR, 645000ATR, 654100ATR, 613301ATB, 613302ATB, 616401ATB, 619101ATB, 624201ATB, 624202ATB, 624203ATB, 627301ATB, 630300ATB, 630400ATB, 630500ATB, 630600ATB, 635600ATB, 635700ATB, 675500ATB, 639601ATB, 641800ATR, 641900ATR, 642000ATR, 645301ATB, 648400ATB, 648500ATB, 648600ATB, 649900ATR, 650000ATR, 650100ATR |
| DM medications and statins | 664600ATB, 664700ATB, 664800ATB, 671800ATR, 673800ATR, 671900ATR, 672000ATR, 672100ATR, 672500ATR, 672600ATR, 672700ATR, 672800ATR, 672900ATR, 673000ATR, 683300ATR, 683400ATR |
| MAOi | 196701ATB, 226401ATB |
| NaSSA | 196201ATD, 196201ATB, 196202ATB, 196202ATD, 196204ATB, 196204ATD |
| NRI | 428101ATB, 428102ATR, 428103ATR |
| SARI | 242901ACH, 242901ATB, 242902ATB, 242903ATR |
| SNRI | 247502ACR, 247502ATR, 247504ACR, 247504ATR, 355801ACH, 355802ACH, 355803ACH, 495501ACE, 4995501ACH, 495501ATB, 499501ATE, 495502ACE, 499502ACH, 499502ATB, 495502ATE, 626401ATR, 626402ATR, 687601ATR, 687602ATR, 687701ATR, 687702ATR |
| SSRIs | 36301ACH, 136302ACH, 161501ACH, 161501ATB, 161502ACH, 161502ATB, 161502ATD, 209301ATB, 209302ATB, 209304ATR, 209305ATR, 227001ATB, 227002ATB, 227003ATB, 495501ACE, 495501ATE, 495502ACE, 495502ATE, 107501ATB, 107502ATB, 107504ATB, 162501ATB, 162502ATB, 173701ATB, 247502ACR, 247504ACR, 428301ATB, 474801ATB, 474802ATB, 474803ATB, 474804ATB, 149203ATB, 149204ATB, 108002ATB, 196201ATD, 196202ATB, 196202ATD, 196204ATB, 196204ATD, 196701ATB, 613101ATB |
| TCA | 149203ATB, 149204ATB, 107501ATB, 107502ATB, 107504ATB, 108002ATB, 136301ACH, 136302ACH, 173701ATB, 188102ATB, 203401ATB |

Abbreviations: ACEi, angiotensin-converting enzyme inhibitor; ARB, angiotensin receptor blocker; BB, beta-blocker; CCB, calcium

channel blocker; DM, diabetes mellitus; TCA, Tricyclic antidepressants; SSRI, selective serotonin reuptake inhibitors;

SNRI, serotonin and norepinephrine reuptake inhibitors; NRI, norepinephrine reuptake inhibitor; NaSSA, noradrenergic and specific

serotonergic antidepressant; MAOi, monoamine oxidase inhibitor

**Table S3:** Specific code of individualized psychotherapy.

| **Code** | **Description** |
| --- | --- |
| NN001 | Individual psychotherapy below 10 minutes |
| NN002 | Individual psychotherapy 10-20 minutes |
| NM003 | Individual Psychotherapy 20-30 minutes |
| NM004 | Individual Psychotherapy 30-40 minutes |
| NM005 | Individual Psychotherapy over 40 minutes |
| NN0021 | Group Psychotherapy |
| NN0021 | Supportive Expressive Group Psychotherapy |
| NN0022 | Dynamic Interactive Group Psychotherapy |
| NN023 | Psychodrama |
| NN0031 | Family therapy, Individual |
| NN0032 | Family therapy, Group |
| NN040 | Occupational or Recreation Therapy |
| NN050 | Nacrosynthesis |
| NN061 | Cognitive Behavioral Therapy, Individual |
| NN062 | Cognitive Behavioral Therapy, Group |
| NN071 | Electroconvulsive Therapy, Simple |
| NN072 | Electroconvulsive Therapy, Modified |
| NN081 | Continuous Sleep Treatment, Electro |
| NN082 | Drug Induced Sleep Treatment |
| NN083 | Sleep Treatment with Anesthesia |
| NN090 | Psychiatric Rehabilitation |
| NN100 | Psychiatric Emergency Treatment |
| NN111 | Psychiatric Social work, Individual history taking |
| NN112 | Psychiatric Social Work, Social work guidance |
| NN113 | Psychiatric Social Work, Social Investigation |
| NN114 | Psychiatric Social Work, Home Visiting |

**Table S4:** Detailed baseline characteristics of the patients before matching.

| **Variables** | **Total (N=16756)** | **Non-PSY (N=8915)** | | **PSY (N=7841)** | ***SMD*** |  |
| --- | --- | --- | --- | --- | --- | --- |
| Age | 67.3 ± 12.9 | 68.3 ± 12.5 | | 66.2 ± 13.3 | 0.339 |  |
| Sex | 8611 (51.4) | 4436 (49.8) | | 4175 (53.3) | 0.015 |  |
| ESKD ~ Depression (Year) | 1.8 ± 2.5 | 1.9 ± 2.6 | | 1.7 ± 2.4 | 0.137 |  |
| Interval of Follow up period | 4.5 ± 3.5 | 4 ± 3.3 | | 5.2 ± 3.6 | 0.227 |  |
| Income level |  |  | |  | 0.087 |  |
| Quantile 1 | 4886 (29.2) | 2691 (30.2) | | 2195 (28.0) |  |  |
| Quantile 2 | 2117 (12.6) | 1124 (12.6) | | 993 (12.7) |  |  |
| Quantile 3 | 3300 (19.7) | 1758 (19.7) | | 1542 (19.7) |  |  |
| Quantile 4 | 6453 (38.5) | 3342 (37.5) | | 3111 (39.7) |  |  |
| Residential area |  |  | |  | 0.070 |  |
| Rural | 8655 (51.6) | 4702 (52.7) | | 3953 (50.4) |  |  |
| Urban | 8101 (48.4) | 4213 (47.3) | | 3888 (49.6) |  |  |
| Myocardial infarction | 3437 (20.5) | 1872 (21.0) | | 1565 (20.0) | 0.047 |  |
| Stroke | 8098 (48.3) | 4625 (51.9) | | 3473 (44.3) | 0.026 |  |
| MACE | 9440 (56.3) | 5307 (59.5) | | 4133 (52.7) | 0.152 |  |
| Amputation | 7 (0.0) | 4 (0.0) | | 3 (0.0) | 0.138 |  |
| Liver cirrhosis | 1609 (9.6) | 833 (9.3) | | 776 (9.9) | 0.003 |  |
| Atrial fibrillation | 2743 (16.4) | 1540 (17.3) | | 1203 (15.3) | 0.019 |  |
| Diabetes mellitus | 15138 (90.3) | 8144 (91.4) | | 6994 (89.2) | 0.052 |  |
| Hypertension | 16446 (98.2) | 8801 (98.7) | | 7645 (97.5) | 0.073 |  |
| Alcohol abuse | 121 (0.7) | 43 (0.5) | | 78 (1.0) | 0.090 |  |
| Drug abuse | 2376 (14.2) | 1229 (13.8) | | 1147 (14.6) | 0.060 |  |
| Year of ESKD diagnosis |  |  | |  | 0.011 |  |
| 2004-2009 | 4873 (29.1) | 2505 (28.1) | | 2368 (30.2) |  |  |
| 2010-2015 | | 5917 (34.5) | | 3165 (35.5) | 2752 (35.1) |  |
| 2016-2022 | | 5966 (36.4) | | 3245 (36.4) | 2721 (34.7) |  |
| Charlson comorbidity | | 6.9±3.2 | | 7.1±3.2 | 6.7±3.1 | 0.167 |
| Myocardial infarction | | 1604 (9.6) | | 884 (9.9) | 720 (9.2) | 0.148 |
| Congestive heart failure | | 5420 (32.4) | | 3068 (34.4) | 2352 (30.0) | 0.025 |
| Peripheral vascular disease | | 5487 (32.8) | | 3037 (34.1) | 2450 (31.3) | 0.095 |
| Cerebrovascular disease | | 7309 (43.6) | | 4243 (47.6) | 3066 (39.1) | 0.060 |
| Dementia | | 4215 (25.2) | | 2420 (27.2) | 1795 (22.9) | 0.172 |
| Chronic pulmonary disease | | 7897 (47.1) | | 4197 (47.1) | 3700 (47.2) | 0.098 |
| Rheumatologic disease | | 1713 (10.2) | | 926 (10.4) | 787 (10.0) | 0.002 |
| Peptic ulcer disease | | 7229 (43.1) | | 3737 (41.9) | 3492 (44.5) | 0.012 |
| Mild liver disease | | 7269 (43.4) | | 3884 (43.6) | 3385 (43.2) | 0.053 |
| Diabetes without chronic complication | | 10790 (64.4) | | 5881 (66.0) | 4909 (62.6) | 0.008 |
| Diabetes with chronic complication | | 8852 (52.8) | | 4914 (55.1) | 3938 (50.2) | 0.070 |
| Hemiplegia, paraplegia | | 1312 (7.8) | | 880 (9.9) | 432 (5.5) | 0.098 |
| Renal disease | | 15756 (100.0) | | 8915 (100.0) | 7841 (100.0) | 0.164 |
| Any malignancy including leukemia and lymphoma | | 2925 (17.5) | | 1618 (18.2) | 1307 (16.7) | 0.012 |
| Moderate, severe liver disease | | 397 (2.4) | | 195 (2.2) | 202 (2.6) | 0.039 |
| Metastatic solid tumor | | 408 (2.4) | | 249 (2.8) | 159 (2.0) | 0.026 |
| AIDS | | 36 (0.2) | | 22 (0.3) | 14 (0.2) | 0.050 |

Abbreviations; AIDS, Acquired Immunodeficiency Syndrome; ESKD, end-stage kidney disease; MACE, major adverse cardiovascular events; SMD, standard mean difference

**Table S5:** Detailed baseline characteristics of patients after matching.

| **Variables** | **Total**  **(N=6,372)** | **Non-PSY (N=7841)** | **PSY (N=7841)** | ***SMD*** |
| --- | --- | --- | --- | --- |
| Age | 66.9 ± 12.9 | 67.6 ± 12.5 | 66.2 ± 13.3 | 0.153 |
| Sex | 8221 (52.4) | 4046 (51.6) | 4175 (53.3) | 0.019 |
| ESRD ~ Depression (Year) | 1.8 ± 2.6 | 2 ± 2.7 | 1.7 ± 2.4 | 0.069 |
| Interval of Follow up period | 4.7 ± 3.5 | 4.1 ± 3.3 | 5.2 ± 3.6 | 0.186 |
| Income level |  |  |  | 0.046 |
| Quantile 1 | 4440 (28.3) | 2245 (28.6) | 2195 (28) |  |
| Quantile 2 | 1996 (12.7) | 1003 (12.8) | 993 (12.7) |  |
| Quantile 3 | 3091 (19.7) | 1549 (19.8) | 1542 (19.7) |  |
| Quantile 4 | 6155 (39.3) | 3044 (38.8) | 3111 (39.7) |  |
| Residential area |  |  |  | 0.033 |
| Rural | 8010 (51.1) | 4057 (51.7) | 3953 (50.4) |  |
| Urban | 7672 (48.9) | 3784 (48.3) | 3888 (49.6) |  |
| Myocardial infarction | 3184 (20.3) | 1619 (20.7) | 1565 (20) | 0.027 |
| Stroke | 7208 (46) | 3735 (47.6) | 3473 (44.3) | 0.017 |
| MACE | 8518 (54.3) | 4385 (55.9) | 4133 (52.7) | 0.067 |
| Amputation | 7 (0) | 4 (0.1) | 3 (0) | 0.065 |
| Liver cirrhosis | 1514 (9.7) | 738 (9.4) | 776 (9.9) | 0.006 |
| Atrial fibrillation | 2484 (15.8) | 1281 (16.3) | 1203 (15.3) | 0.016 |
| Diabetes mellitus | 14112 (90) | 7118 (90.8) | 6994 (89.2) | 0.027 |
| Hypertension | 15373 (98) | 7728 (98.6) | 7645 (97.5) | 0.053 |
| Alcohol abuse | 121 (0.8) | 43 (0.6) | 78 (1) | 0.078 |
| Drug abuse | 2216 (14.1) | 1069 (13.6) | 1147 (14.6) | 0.052 |
| Year of ESKD diagnosis |  |  |  | 0.003 |
| 2004-2009 | 4650 (29.6) | 2282 (29.1) | 2368 (30.2) |  |
| 2010-2015 | 5489 (35.0) | 2737 (34.9) | 2752 (35.1) |  |
| 2016-2022 | 5544 (35.4) | 2823 (36.0) | 2721 (34.7) |  |
| Charlson comorbidity | 6.8 ± 3.1 | 6.9 ± 3.1 | 6.7 ± 3.1 | 0.108 |
| Myocardial infarction | 1490 (9.5) | 770 (9.8) | 720 (9.2) | 0.057 |
| Congestive heart failure | 4881 (31.1) | 2529 (32.3) | 2352 (30) | 0.022 |
| Peripheral vascular disease | 5028 (32.1) | 2578 (32.9) | 2450 (31.3) | 0.048 |
| Cerebrovascular disease | 6417 (40.9) | 3351 (42.7) | 3066 (39.1) | 0.035 |
| Dementia | 3786 (24.1) | 1991 (25.4) | 1795 (22.9) | 0.074 |
| Chronic pulmonary disease | 7354 (46.9) | 3654 (46.6) | 3700 (47.2) | 0.058 |
| Rheumatologic disease | 1633 (10.4) | 846 (10.8) | 787 (10) | 0.012 |
| Peptic ulcer disease | 6850 (43.7) | 3358 (42.8) | 3492 (44.5) | 0.025 |
| Mild liver disease | 6774 (43.2) | 3389 (43.2) | 3385 (43.2) | 0.035 |
| Diabetes without chronic complication | 9956 (63.5) | 5047 (64.4) | 4909 (62.6) | 0.001 |
| Diabetes with chronic complication | 8107 (51.7) | 4169 (53.2) | 3938 (50.2) | 0.037 |
| Hemiplegia, paraplegia | 881 (5.6) | 449 (5.7) | 432 (5.5) | 0.059 |
| Renal disease | 12656 (80.7) | 6305 (80.4) | 6351 (81) | 0.008 |
| Any malignancy including leukemia and lymphoma | 2668 (17) | 1361 (17.4) | 1307 (16.7) | 0.015 |
| Moderate, severe liver disease | 384 (2.5) | 182 (2.3) | 202 (2.6) | 0.018 |
| Metastatic solid tumor | 327 (2.1) | 168 (2.1) | 159 (2) | 0.017 |
| AIDS | 35 (0.2) | 21 (0.3) | 14 (0.2) | 0.007 |

Abbreviations; AIDS, Acquired Immunodeficiency Syndrome; ESKD, end-stage kidney disease; MACE, major adverse cardiovascular events; SMD, standard mean difference

**Table S6:** Cumulative incidence and hazard ratio of death before and after matching.

| **Group-before matching** | **Cumulative incidence (%)** | | | **HR (95% CI)** | ***p-value*** |
| --- | --- | --- | --- | --- | --- |
|  | **1Y** | **3Y** | **5Y** |  |  |
| Non-PSY | 16.6 | 30.4 | 38.2 | 1 |  |
| PSY | 7 | 18.3 | 25.8 | 0.60(0.57-0.64) | <.0001 |
| Group-after matching | **Cumulative incidence (%)** | | | HR (95% CI) | *p-value* |
|  | 1Y | 3Y | 5Y |  |  |
| Non-PSY | 15.1 | 28.7 | 36.3 | 1 |  |
| PSY | 7 | 18.3 | 25.8 | 0.65(0.61-0.68) | <.0001 |

Abbreviations; CI, confidence intervals; HR, hazard ratio

**Table S7:** Risk of death with landmark analysis and without landmark analysis (univariate analysis).

|  |  | **HR (95% CI)** | ***p value*** |
| --- | --- | --- | --- |
| Landmark-No |  | 0.65 (0.61-0.68) | <0.001 |
| Landmark-Yes | 6 month | 0.70 (0.66-0.75) | <0.001 |
|  | 1 year | 0.76 (0.71-0.81) | <0.001 |
|  | 2 year | 0.72 (0.66-0.78) | <0.001 |
|  | 3 year | 0.66 (0.59-0.74) | <0.001 |

Abbreviations; CI, confidence intervals; HR, hazard ratio

**Table S8:** Type of prescribed antidepressants between two group (before and after matching).

|  | **Before matching** |  |  | **After Matching** |  |  |
| --- | --- | --- | --- | --- | --- | --- |
|  | Non PSY (8915) | PSY (7841) | *P* | Non PSY (7841) | PSY (7841) | *P* |
| SSRI | 1715 (19.2) | 2962 (37.8) | <0.001 | 2002 (25.5) | 2962 (37.8) | <0.001 |
| SNRI | 732 (8.2) | 632 (8.1) | 0.005 | 806 (10.3) | 632 (8.1) | 0.024 |
| SARI | 630 (7.1) | 1002 (12.8) | <0.001 | 747 (9.5) | 1002 (12.8) | <0.001 |
| NaSSa | 155 (1.7) | 454 (5.8) | <0.001 | 176 (2.2) | 454 (5.8) | <0.001 |
| TCA | 4071 (45.7) | 2615 (33.4) | <0.001 | 4586 (58.5) | 2615 (33.4) | <0.001 |
| NRI | 63 (0.7) | 81 (1) | 0.132 | 76 (1) | 81 (1) | 0.226 |
| MAOi | 0 (0) | 6 (0.1) | 0.014 | 1 (0) | 6 (0.1) | 0.039 |

Abbreviations: TCA, Tricyclic antidepressants; SARI, serotonin antagonist and reuptake inhibitor; SSRI, selective serotonin reuptake inhibitors; SNRI, serotonin and norepinephrine reuptake inhibitors; NRI, norepinephrine reuptake inhibitor; NaSSA, noradrenergic and specific serotonergic antidepressant; MAOi, monoamine oxidase inhibitor

**Table S9:** Type of prescribed selective serotonin reuptake inhibitors (SSRIs) between two groups.

|  | **Before matching** | |  | **After Matching** | |  |
| --- | --- | --- | --- | --- | --- | --- |
|  | Non PSY (2002) | PSY (2962) | *P* | Non PSY (1715) | PSY (2962) | *p* |
| Long QT | 1304 (65.1) | 1524 (51.4) | <0.001 | 1140 (66.5) | 1524 (51.4) | <0.001 |
| Short QT | 698 (34.9) | 1438 (48.6) |  | 575 (33.5) | 1438 (48.6) |  |

Long QT: SSRIs with higher prolong QT potential: citalopram and esitalopram

Short QT: SSRIs with lower prolong QT potential: sertraline, paroxetine, fluoxetine, fluvoxamine

**Table S10:** Type of prescribed selective serotonin reuptake inhibitors (SSRIs) and clinical outcomes.

|  | Before matching | | | | After Matching | | | |
| --- | --- | --- | --- | --- | --- | --- | --- | --- |
|  | All-cause death | P int | Cardiovascular death | P int | All-cause death | P int | Cardiovascular death | P int |
| Long QT | 0.70 (0.64–0.78) | 0.08 | 0.79 (0.68–0.91) | 0.09 | 0.71 (0.65–0.78) | 0.03 | 0.81 (0.70–0.93) | 0.07 |
| Short QT | 0.66 (0.59–0.74) |  | 0.73 (0.63–0.85) |  | 0.64 (0.58–0.71) |  | 0.74 (0.63–0.87) |  |

Reference; Non PSY group.

Long QT: SSRIs with higher prolong QT potential: citalopram and esitalopram

Short QT: SSRIs with lower prolong QT potential: sertraline, paroxetine, fluoxetine, fluvoxamine

**Figure S1.** Graphic depiction of landmark analysis.


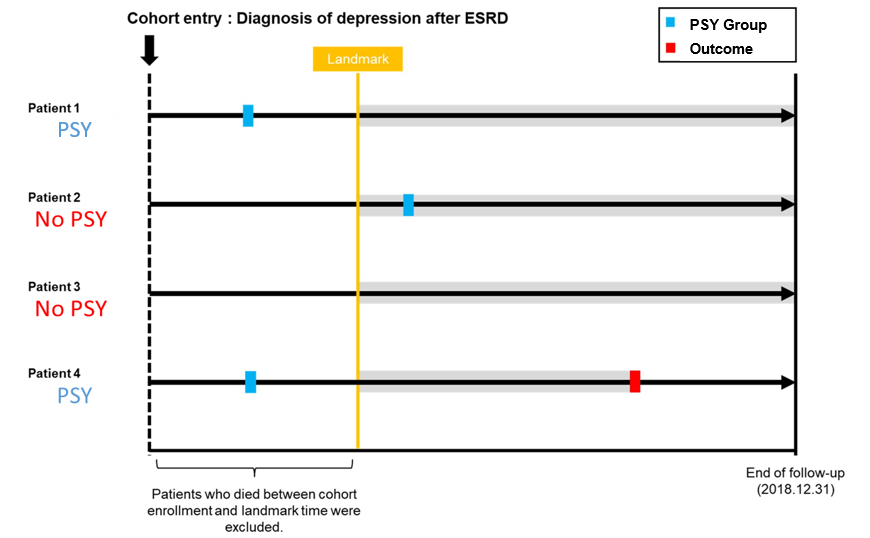


Blue squares represent patients prescribed antidepressants by psychiatrists and red squares represent outcomes. To ensure that all patients were followed for at least 1 year, we set the last cohort entry date as December 31, 2018. Patients who died between the cohort entry and landmark dates were excluded. Patient 1 represents patients received antidepressants by psychiatrists within the landmark period (categorized in the PSY group). Patient 2 represents patients prescribed antidepressants by psychiatrists after the landmark period (categorized in non-PSY group). Patient 3 represents patients who were not prescribed antidepressants by psychiatrists (categorized in the non-PSY group). Patient 4 represents patients who were prescribed antidepressants by psychiatrists between the cohort entry and landmark times and who developed a study outcome after the landmark period (categorized in the PSY group).

Figure S2. Standard mean difference before and after matching.


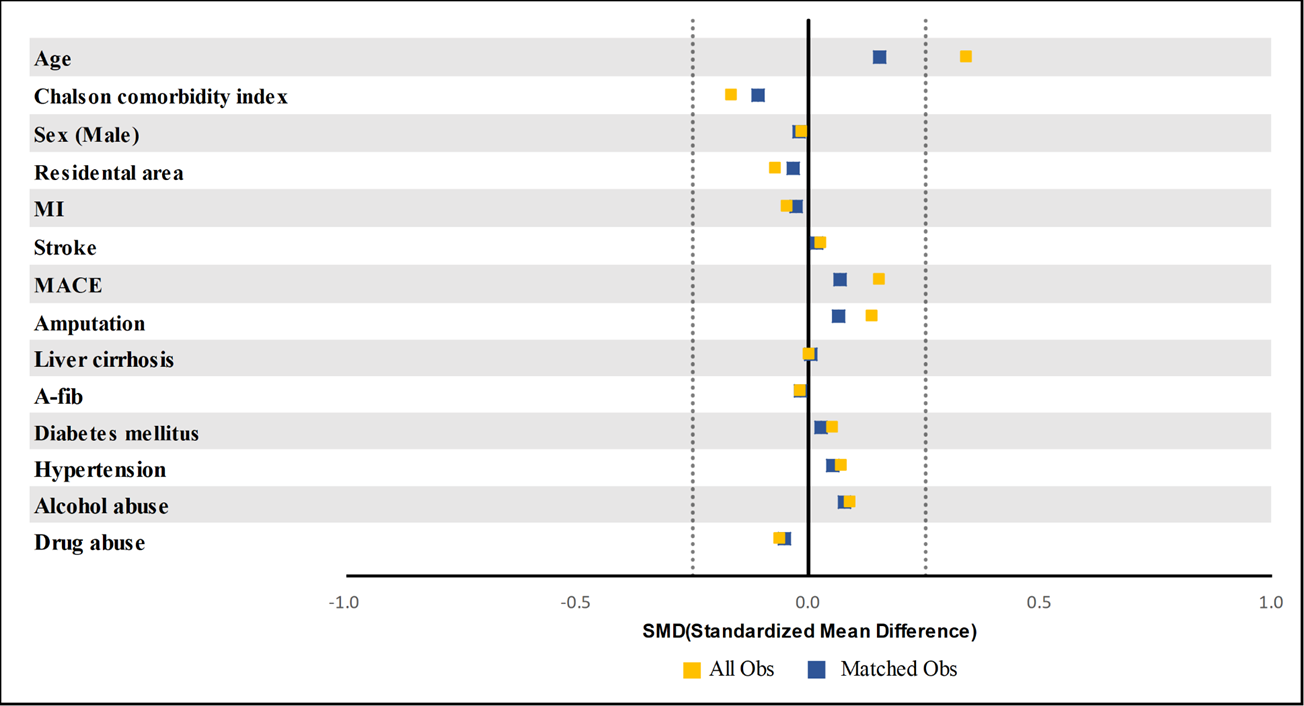


**Figure S3**. Kaplan-Meier curve for all cause death (landmark analysis).

1. 6 months


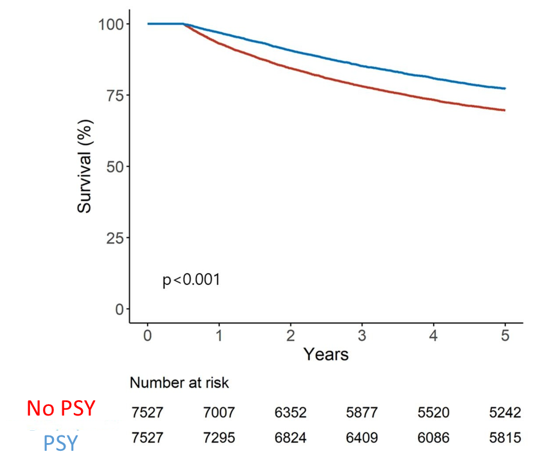


b) 12 months


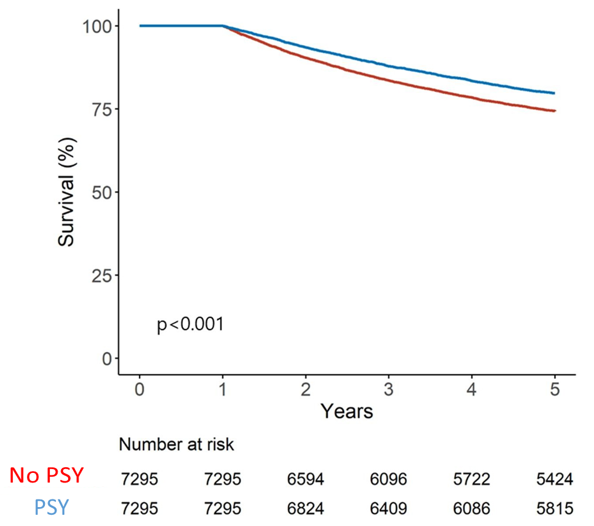


1. 24 months


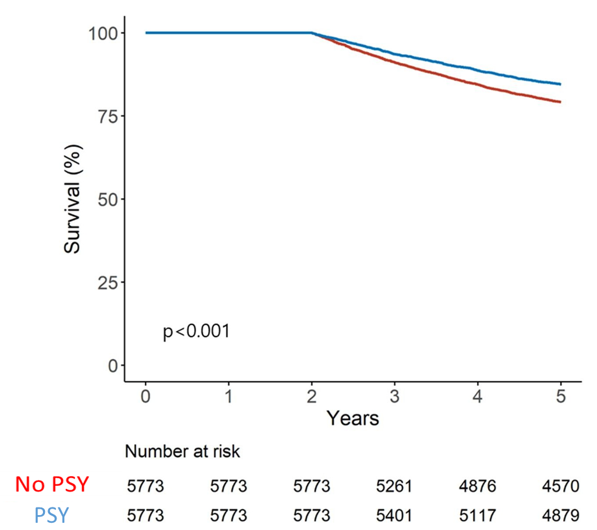


1. 36 months


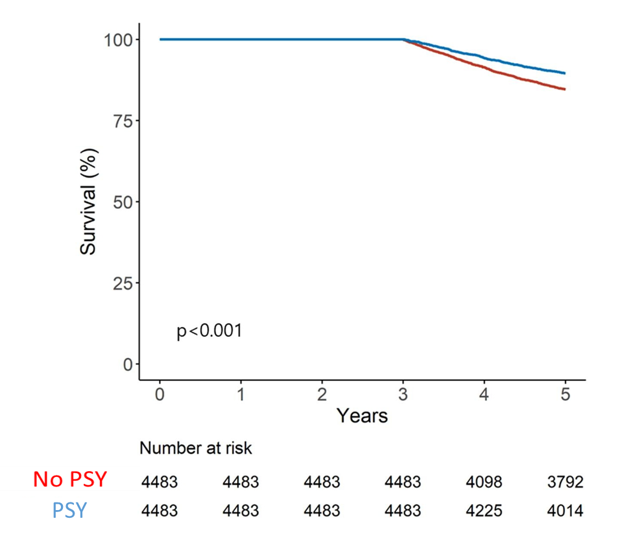

Supplement: sfaf374_Supplemental_File [file sfaf374_supplemental_file.docx]
